# Incorporation of copper ions into crystals of T2 copper-depleted laccase from *Botrytis aclada*

**DOI:** 10.1107/S2053230X1502052X

**Published:** 2015-11-18

**Authors:** E. M. Osipov, K. M. Polyakov, T. V. Tikhonova, R. Kittl, P.V. Dorovatovskii, S. V. Shleev, V. O. Popov, R. Ludwig

**Affiliations:** a Laboratory of Enzyme Engineering, A. N. Bach Institute of Biochemistry, Leninsky pr. 33, Moscow 119071, Russian Federation; b Engelhardt Institute of Molecular Biology, Vavilova str. 32, Moscow 119991, Russian Federation; c Department of Food Science and Technology, BOKU – University of Natural Resources and Life Sciences, Muthgasse 18, 1190 Wien, Austria; d RSC ‘Kurchatov Institute’, Acad. Kurchatov sq. 1, Moscow 123182, Russian Federation

**Keywords:** laccase, copper(I) complex, full copper content

## Abstract

Laccases belong to the class of multicopper oxidases catalyzing the oxidation of phenols accompanied by the reduction of molecular oxygen to water without the formation of hydrogen peroxide. The activity of laccases depends on the number of Cu atoms per enzyme molecule. The structure of type 2 copper-depleted laccase from *Botrytis aclada* has been solved previously. With the aim of obtaining the structure of the native form of the enzyme, crystals of the depleted laccase were soaked in Cu^+^- and Cu^2+^-containing solutions. Copper ions were found to be incorporated into the active site only when Cu^+^ was used. A comparative analysis of the native and depleted forms of the enzymes was performed.

## Abstract 2

The restoration of the native form of laccase from *B. aclada* from the type 2 copper-depleted form of the enzyme was investigated. Copper ions were found to be incorporated into the active site after soaking the depleted enzyme in a Cu^+^-containing solution.

## 1. Introduction   

Laccases (EC 1.10.3.2; benzenediol:oxygen oxidoreductases) belong to the class of multicopper oxidases catalyzing the oxidation of phenols accompanied by the reduction of molecular oxygen to water. Laccases are composed of two or three structurally similar cupredoxin-like domains. These enzymes have been well studied both by biochemical (Baldrian, 2006 ▸) and structural (Hakulinen & Rouvinen, 2015 ▸) methods. Owing to their broad substrate specificity and the formation of non­toxic products, laccases are of interest in biotechnology (Xu, 2002 ▸).

The active site of laccases comprises four Cu atoms classified into three types depending on their spectroscopic characteristics (Solomon *et al.*, 1996 ▸). The type 1 copper ion can be detected in the ESR spectrum and gives a characteristic absorption at around 600 nm. This ion has a trigonal planar coordination through the N^∊^ atoms of two histidines and the S atom of one cysteine. Type 2 and type 3 copper ions form a T2/T3 cluster responsible for the reduction of molecular oxygen to water. The type 2 copper ion is detected in the ESR spectrum and does not show absorption in the optical region. Two type 3 copper ions are antiferromagnetically coupled through an oxygen-containing ligand and consequently do not exhibit an ESR signal, but show an absorption maximum at around 330 nm. The type 2 copper ion adopts a square-planar coordination geometry with two histidines and one or two oxygen-containing ligands. Each type 3 copper ion is coordinated in a tetrahedral geometry by three histidines and one oxygen-containing ligand, through which it is linked to another type 3 copper ion.

The activity of laccase preparations depends on the number of Cu atoms per enzyme molecule. Thus, one way of investigating laccases is to study enzyme preparations in which the copper ions have been partially removed. The type 2 copper ion is more easily removed from the laccase molecule compared with the other copper ions (Malkin *et al.*, 1969 ▸). On the one hand, procedures have been developed for the preparation of inactive type 2 copper-depleted forms of the enzyme based on treatment with copper chelators in the presence of reducing agents (Reinhammar & Oda, 1979 ▸; Koroleva *et al.*, 2001 ▸). On the other hand, the partial loss of Cu atoms from the active site can occur spontaneously during the production and storage of laccases.

In some X-ray diffraction structures of laccases which had not been subjected to a copper-chelation procedure, the type 2 copper ion was either absent or had partial occupancy (Glazunova *et al.*, 2015 ▸; Ducros *et al.*, 1998 ▸; Osipov *et al.*, 2014 ▸). The restoration of depleted forms of laccases with a simultaneous increase in their activity is accomplished by treatment of the enzyme with copper salts. The insertion of a type 2 copper ion into fungal, plant and bacterial laccases using Cu^+^ ions has been described in the literature (Malkin *et al.*, 1969 ▸; Reinhammar & Oda, 1979 ▸; Koroleva *et al.*, 2001 ▸). The X-ray crystal structure of the fungal type 2 copper-depleted laccase from *Coriolopsis caperata* showed that a copper ion was inserted into the active site only when Cu^+^ ions were used, whereas the use of Cu^2+^ ions did not give the desired result (Glazunova *et al.*, 2015 ▸). However, it was demonstrated that the incorporation of a type 2 copper ion into the bacterial laccase from *Bacillus subtilis* occurs in the presence of either Cu^+^ or Cu^2+^ ions (Durão *et al.*, 2008 ▸).

The laccase from the ascomycete *Botrytis aclada* has previously been isolated and characterized biochemically (Kittl *et al.*, 2012 ▸). The structures of this enzyme (hereafter referred to as T2D) and its L499M mutant were determined at 1.7 Å resolution (Osipov *et al.*, 2014 ▸). Although the laccase was not subjected to a depletion procedure, the type 2 copper ion was absent in both structures and the residue His429, *i.e.* one of the two histidines that are involved in the coordination of the type 2 copper ion, points towards Cu3_α_.

The aim of the study presented here is to determine the structure of *B. aclada* laccase containing the complete set of copper ions. For this purpose, crystals of the type 2 copper-depleted form of *B. aclada* laccase were soaked in solutions containing Cu^+^ or Cu^2+^ ions. Restoration of the native form of the enzyme was only observed in the experiment employing Cu^+^ ions.

## 2. Materials and methods   

### 2.1. Purification and crystallization of laccase   

Laccase from *B. aclada* (Table 1 ▸) was recombinantly expressed in the yeast *Pichia pastoris* and purified as described by Kittl *et al.* (2012 ▸). Attempts to obtain crystals of the native form of laccase failed. Therefore, the enzyme was deglycosyl­ated (Osipov *et al.*, 2014 ▸). The crystallization conditions have been described in detail in a previous study (Osipov *et al.*, 2014 ▸). Crystals were grown by the vapour-diffusion technique. A 1.8 *M* solution of ammonium sulfate in water was used as the reservoir solution. The protein solution consisted of 20 mg ml^−1^ protein in 25 m*M* sodium acetate buffer pH 5.0. A 2 µl drop composed of equal volumes of the protein and reservoir solutions was used (Table 2 ▸). Crystals appeared within 3 d and reached maximum dimensions of 0.2 × 0.1 × 0.05 mm in one month.

### 2.2. Preparation of complexes of T2D with Cu^+^ and Cu^2+^   

The complexes were obtained by soaking crystals of the enzyme in reservoir solution containing 0.6 m*M* (saturated solution) CuCl (T2D+Cu^+^ complex) or 10 m*M* CuSO_4_ (T2D+Cu^2+^ complex). The soaking times were 10 min and 1 d, respectively.

### 2.3. Data collection and processing   

X-ray data sets for T2D+Cu^+^ and T2D+Cu^2+^ were collected on the K4.4e beamline at the Belok station at the Kurchatov synchrotron-radiation source at 100 K under a nitrogen flow at a wavelength of 0.98 Å using a Rayonix SX165 detector. Before X-ray data collection, the crystals were placed for 5 s in reservoir solution supplemented with 20%(*v*/*v*) glycerol. The X-ray data sets were processed using the *XDS* package (Kabsch, 2010*a* ▸,*b* ▸). The data-collection statistics are summarized in Table 3 ▸.

### 2.4. Structure solution and refinement   

The crystals of the complexes were isomorphous to the crystals of T2D. The structures were refined with *REFMAC*5 (Murshudov *et al.*, 2011 ▸). All crystallographic calculations were carried out using the *CCP*4 suite (Winn *et al.*, 2011 ▸). During the refinement of the copper ions with full occupancy, the *F*_o_ − *F*_c_ difference map contained essential peaks in the region of the copper ions and the *B* factors of the copper ions exceeded the *B* factors of the ligands. Therefore, in the final stages of refinement the occupancies of the copper ions were refined manually. Manual correction of the occupancies was followed by *B*-factor refinement. In the final model, the *B* factors of the copper ions were approximately equal to the *B* factors of the ligands, and the *F*_o_ − *F*_c_ difference map did not contain essential peaks in the region of the copper ions. Visual inspection and manual rebuilding of the models was carried out using the *Coot* interactive graphics program (Emsley *et al.*, 2010 ▸). Water molecules were manually added to the structures based on analysis of the difference electron-density maps. The quality of the structures was evaluated with *SFCHECK* (Vaguine *et al.*, 1999 ▸) and *PROCHECK* (Laskowski *et al.*, 1993 ▸). The figures were drawn with *CCP*4*mg* (McNicholas *et al.*, 2011 ▸). *MolProbity* (Chen *et al.*, 2010 ▸) was used for Ramachandran analysis. Structure-solution and refinement statistics are summarized in Table 4 ▸.

## 3. Results and discussion   

The structure of T2D+Cu^2+^ was solved at 1.8 Å resolution. The crystals of T2D+Cu^2+^ have the same qualities as the initial crystals of T2D. Since the structure of T2D+Cu^2+^ is almost identical to that of T2D, it was not deposited in the PDB. The structures of T2D and T2D+Cu^2+^ superimposed with an r.m.s.d. of 0.09 Å using the coordinates of 539 equivalent C^α^ atoms. Soaking T2D crystals for 24 h in a solution containing Cu^2+^ ions at high concentrations did not lead to the insertion of a copper ion into the T2/T3 cluster.

The complexes were obtained using crystals grown in the same drop. During the preparation of the T2D+Cu^+^ crystals the quality of the crystals visually deteriorated despite the low concentration of Cu^+^ ions and the short period of soaking. The structure of T2D+Cu^+^ was solved at 2.3 Å resolution. The reduction in the diffraction limit was accompanied by an increase in the mosaicity (0.38° for the crystals of T2D+Cu^+^ *versus* 0.25° for the crystals of T2D). Superimposition of the T2D+Cu^+^ and T2D structures using the coordinates of 525 equivalent C^α^ atoms gave an r.m.s.d. of 0.16 Å. Residues 1–14 (the numbering given is according to the T2D structure) and residues 405–408 were not located in the electron-density map for T2D+Cu^+^. The latter residues are also not observed in the electron-density maps for T2D and T2D+Cu^2+^.

**Figure 1:**
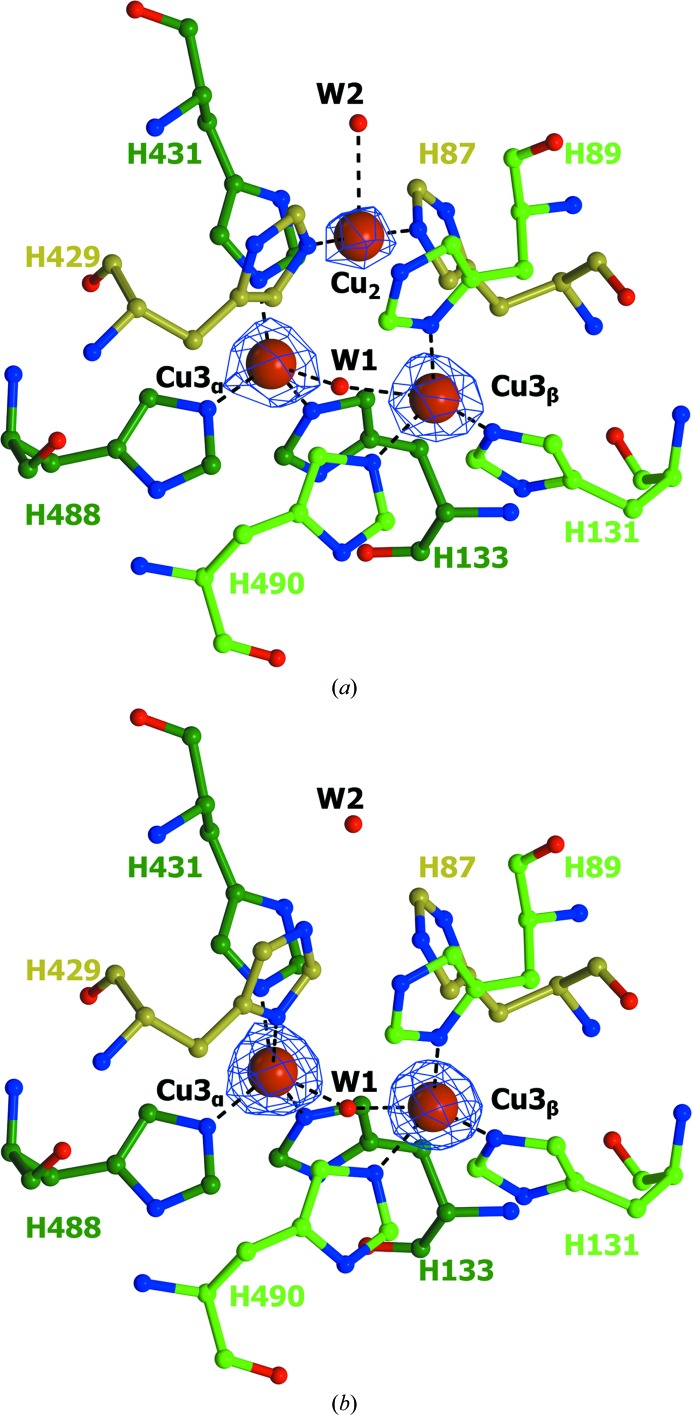
Structures of the T2/T3 copper sites in (*a*) T2D+Cu^+^ and (*b*) T2D. The amino-acid residues are shown as ball-and-stick models and are coloured according to atom type. The C atoms of the His residues coordinated to the Cu3_α_, Cu3_β_ and Cu2 ions are shown in dark green, light green and gold, respectively. The oxygen ligands and copper ions are shown as red and orange spheres, respectively. The 2*F*_o_ − *F*_c_ electron-density maps (at the 6σ level) for copper ions are shown in blue. Coordination bonds are indicated by black dashed lines.

The main differences in the structures of T2D+Cu^+^ and T2D are related to the location of a copper ion in the type 2 site and the orientation of the side chain of His429 (Fig. 1 ▸). It should be noted that in the structure of T2D Cu2 is completely absent and the sum of occupancies of the copper ions in T2D (2.6) is almost equal to the content of copper ions per molecule for the enzyme in solution as determined by mass spectrometry (Osipov *et al.*, 2014 ▸). In the T2D+Cu^+^ structure the type 2 copper ion has a square-planar coordination formed by the His87 N^∊^ and His429 N^∊^ atoms and the O atom of a water molecule (Table 5 ▸). This situation is typical for all laccases containing type 2 copper ions. In the T2D structure His429 is coordinated to Cu3_α_ by the N^δ^ atom (Osipov *et al.*, 2014 ▸). Thus, the removal of type 2 copper ions could be associated with changes in the orientation of the side chain of His429. It should be noted that the occupancy of the type 2 copper ion is lower than the occupancy of the type 3 copper ions. Inter­atomic distances, *B* factors and occupancies for copper ions in the T2/T3 cluster and their respective ligands are shown in Table 5 ▸. The lower occupancy of the type 2 copper ion is also clearly visible in the electron-density map of T2D+Cu^+^ (Fig. 1 ▸ *a*).

In the structures of T2 copper-depleted laccases from *Coprinus cinereus* (Ducros *et al.*, 1998 ▸), *Coriolopsis gallica* (De la Mora *et al.*, 2012 ▸) and *Trametes hirsuta* (Polyakov *et al.*, 2009 ▸) a histidine residue equivalent to His429 also forms a weak coordination bond to Cu3_α_.

In addition, the electron-density map of the T2D+Cu^+^ complex has a peak in the vicinity of His81 at a distance of 2.1 Å from the N^∊^ atom. This peak was interpreted as a copper ion with an occupancy of 0.2.

It should be noted that the incorporation of copper into the T2 site was unambiguously determined by the X-ray diffraction data. In the study of laccase from *B. aclada* it was shown that the incorporation of a type 2 copper ion only occurs in experiments with Cu^+^ ions. In the case of laccase from *C. caperata*, reconstitution was also observed only with the use of Cu^+^ salts (Glazunova *et al.*, 2015 ▸). Since the active sites of laccases from different organisms have similar structures, it can be suggested that only Cu^+^ ions can be efficiently incorporated into the type 2 copper-depleted active sites of all laccases. This is in good agreement with the fact that Cu^+^ ions are inserted into the type 2 sites of depleted laccases (Malkin *et al.*, 1969 ▸; Reinhammar & Oda, 1979 ▸). It has been shown by ESR spectroscopy and kinetic measurements that both Cu^+^ and Cu^2+^ ions are inserted into the bacterial laccase from *B. subtilis* (Durão *et al.*, 2008 ▸). However, spectroscopic evidence for the incorporation of copper ions into the enzyme was only presented for Cu^+^; the kinetic data show that copper ions are more efficiently inserted as Cu^+^ ion into the laccase from *B. subtilis*.

## Supplementary Material

PDB reference: *Botrytis aclada* laccase, 4x4k

## Figures and Tables

**Table 1:** Macromolecule-production information

Source organism	*B. aclada*
Forward primer 5BAPml1	5′-ATACACGTGCAAGATGAAGTATTTCACAGTCTTTACTGC-3′
Reverse primer 3BAXba1	5′-ATATCTAGACTTAAATTCCAGAATCGTCCTC-3′
Expression vector	pPICZB and pPICZαA
Expression host	*P. pastoris*
Complete amino-acid sequence of the construct produced	MKYFTVFTALTALFAQASASAIPAVRSTLTPRQNTTASCANSATSRSCWGEYSIDTNWYDVTPTGVTREYWLSVENSTITPDGYTRSAMTFNGTVPGPAIIADWGDNLIIHVTNNLEHNGTSIHWHGIRQLGSLEYDGVPGVTQCPIAPGDTLTYKFQVTQYGTTWYHSHFSLQYGDGLFGPLIINGPATADYDEDVGVIFLQDWAHESVFEIWDTARLGAPPALENTLMNGTNTFDCSASTDPNCVGGGKKFELTFVEGTKYRLRLINVGIDSHFEFAIDNHTLTVIANDLVPIVPYTTDTLLIGIGQRYDVIVEANAAADNYWIRGNWGTTCSTNNEAANATGILRYDSSSIANPTSVGTTPRGTCEDEPVASLVPHLALDVGGYSLVDEQVSSAFTNYFTWTINSSSLLLDWSSPTTLKIFNNETIFPTEYNVVALEQTNANEEWVVYVIEDLTGFGIWHPIHLHGHDFFIVAQETDVFNSDESPAKFNLVNPPRRDVAALPGNGYLAIAFKLDNPGSWLLHCHIAWHASEGLAMQFVESQSSIAVKMTDTAIFEDTCANWNAYTPTQLFAEDDSGI

**Table 2:** Crystallization

Method	Vapour diffusion
Plate type	VDX
Temperature (K)	298
Protein concentration (mg ml^−1^)	20
Buffer composition of protein solution	25 m*M* sodium acetate pH 5.0
Composition of reservoir solution	1.8 *M* ammonium sulfate
Volume and ratio of drop	2 µl, 1:1
Volume of reservoir	500 µl

**Table 3:** Data collection and processing Values in parentheses are for the outer shell.

Data set	T2D+Cu^+^	T2D+Cu^2+^
Beamline	Beamline K4.4e, Kurchatov SNC	
Wavelength (Å)	0.98	
Temperature (K)	100	
Detector	Rayonix SX165 CCD	
Rotation range per image (°)	1.0	
Total rotation range (°)	154.0	119.0
Space group	*C*2	
*a*, *b*, *c* (Å)	69.4, 113.1, 79.6	70.1, 113.9, 80.0
α, β, γ (°)	90, 109.0, 90	90, 108.8, 90
Mosaicity (°)	0.37	0.26
Resolution range (Å)	30–2.30 (2.44–2.30)	30–1.83 (1.94–1.83)
Total No. of reflections	81583 (12203)	124585 (16545)
No. of unique reflections	25106 (3913)	50252 (7615)
Completeness (%)	96.7 (94.6)	96.1 (90.5)
Multiplicity	3.3 (3.1)	2.5 (2.2)
〈*I*/σ(*I*)〉	16.3 (2.3)	20.8 (5.1)
*R*_meas_	0.071 (0.60)	0.039 (0.23)
Overall *B* factor from Wilson plot (Å^2^)	37.7	24.4

**Table 4:** Structure solution and refinement Values in parentheses are for the outer shell.

Data set	T2D+Cu^+^	T2D+Cu^2+^
Resolution range (Å)	30–2.30 (2.44–2.30)	30–1.83 (1.94–1.83)
Completeness (%)	97.2	96.3
No. of reflections, working set	23801 (1706)	47673 (3062)
No. of reflections, test set	1292 (111)	2579 (187)
Final *R*_cryst_	0.181 (0.278)	0.162 (0.241)
Final *R*_free_	0.233 (0.347)	0.204 (0.302)
No. of non-H atoms		
Protein	4087	4208
Ion	5	3
Water	155	429
Other	197	197
Total	4444	4837
R.m.s. deviations		
Bonds (Å)	0.011	0.016
Angles (°)	1.62	1.92
Average *B* factors (Å^2^)		
Protein	38.6	23.3
Ion	38.3	21.2
Water	35.8	29.8
Other	58.9	37.7
Ramachandran plot		
Favoured (%)	96.2	96.8
Allowed (%)	3.6	3.2

**Table 5:** Interatomic distances and temperature factors in the T2/T3 clusters of T2D+Cu^+^ and T2D+Cu^2+^ Occupancies for copper ions are given in parentheses.

		T2D+Cu^+^			T2D+Cu^2+^		
			*B* factor (Å^2^)			*B* factor (Å^2^)	
Atom *A*	Atom *B*	*A*–*B* distance (Å)	Atom *A*	Atom *B*	*A*–*B* distance (Å)	Atom *A*	Atom *B*
Cu3_α_	His133 N^∊^	2.06	34.2 (0.8)	31.4	2.12	20.8 (0.8)	19.2
	His431 N^∊^	1.95		28.3	2.04		15.0
	His488 N^∊^	1.91		30.4	2.03		20.0
	His429 N^δ^	—		—	2.8		24.3
	His429 C^δ^	3.35		29.5	—		—
	W1	2.03		26.7	1.90		24.5
Cu3_β_	His89 N^δ^	2.04	30.5 (0.8)	34.6	2.02	22.7 (0.8)	16.0
	His131 N^∊^	2.01		28.6	2.05		19.6
	His490 N^∊^	2.10		33.2	2.11		18.8
	W1	2.75		26.7	2.79		24.5
Cu2	His87 N^∊^	1.96	35.6 (0.7)	32.0	—	—	—
	His429 N^∊^	1.77		29.9	—	—	—
	W1	3.38		26.7	—	—	—
	W2	2.54		35.1	—	—	—
